# Outcomes of Endoscopic Retrograde Cholangiopancreatography in End-Stage Renal Disease Patients Undergoing Hemodialysis: A Systematic Review and Pooled Analysis

**DOI:** 10.3390/jpm12111883

**Published:** 2022-11-10

**Authors:** Tae Young Park, Chang Seok Bang, Jae Hyuk Do, Hyoung Chul Oh

**Affiliations:** 1 Division of Gastroenterology, Chung-Ang University College of Medicine, 102 Heukseok-ro, Dongjak-gu, Seoul 06973, Korea; 2 Department of Internal Medicine, Chuncheon Sacred Heart Hospital, Hallym University College of Medicine, Chuncheon 24253, Korea

**Keywords:** endoscopic retrograde cholangiopancreatography, end-stage renal disease, hemodialysis, adverse events, systematic review

## Abstract

Background/Aims: The adverse events associated with endoscopic retrograde cholangiopancreatography (ERCP) in end-stage renal disease (ESRD) patients undergoing hemodialysis (HD) have not been sufficiently evaluated. This study aimed to review the morbidity and mortality associated with ERCP in ESRD patients on HD using a systematic review and pooled analysis. Methods: A systematic review and pooled analysis were conducted on studies that evaluated the clinical outcomes of ERCP in patients on HD. Random-effect model meta-analyses with subgroup analyses were conducted. The methodological quality of the included publications was evaluated using the risk of bias assessment tool for nonrandomized studies. The publication bias was assessed. Results: A total of 239 studies were identified, and 12 studies comprising 7921 HD patients were included in the analysis. The pooled estimated frequency of bleeding associated with ERCP in HD patients was 5.8% (460/7921). In the subgroup analysis of seven comparative studies, the ERCP-related bleeding rate was significantly higher in HD patients than in non-HD patients (5.5% (414/7544) vs. 1.5% (6734/456,833), OR 3.84; 95% CI 4.26–25.5; *p* < 0.001). The pooled frequency of post-ERCP pancreatitis was 8.3%. The pooled frequency of bowel perforation was 0.3%. The pooled estimated mortality associated with ERCP was 7.1% The publication bias was minimal. Conclusion: This pooled analysis showed that ERCP-related morbidity and mortality are higher in HD patients than in non-dialysis patients.

## 1. Introduction

The prevalence of gallstone disease has been reported to be higher in chronic kidney disease patients and end-stage renal disease (ESRD) patients on hemodialysis (HD) [1,2,3]. As the number of ESRD patients on HD with gallstones has increased, the need for endoscopic retrograde cholangiopancreatography (ERCP) in HD patients has gradually increased [4].

ERCP is one of the most commonly performed therapeutic endoscopies; however, therapeutic ERCP is an invasive procedure [5]. ERCP has an inherent risk of adverse events, including bleeding, post-ERCP pancreatitis, bowel perforation, biliary infection, and cardiopulmonary complications [6]. Although improvements in endoscopic technologies and skills are now making the complicated procedure more possible today, the risk of complications remains a troublesome issue. In addition, the risk of ERCP-related adverse events in HD patients might differ from those in the general population. In ESRD patients undergoing HD, the risk of ERCP-related adverse events can be higher due to patient coagulopathies, including platelet dysfunction and activation of fibrinolysis, uremic conditions, bowel vulnerability, immunocompromised status, and underlying cardiopulmonary diseases [7,8,9]. For these reasons, performing ERCP in ESRD patients on HD has been recognized as a procedure with a high risk of complications, and endoscopists regard it as a burdensome procedure [10].

To date, there have been few relevant studies on ERCP in ESRD patients undergoing HD because of ethical and practical limitations due to procedure-related morbidity and mortality. Therefore, ERCP-related adverse events, including bleeding, post-ERCP pancreatitis, bowel perforation, and mortality, have not been sufficiently evaluated. This study aimed to assess the outcomes of ERCP in ESRD patients on HD and focused on morbidity using a systematic review and pooled analysis.

## 2. Materials and Methods

### 2.1. Study Identification and Selection Criteria

The systematic review and meta-analysis were conducted according to the preferred reporting items for systematic reviews and meta-analyses (PRISMA) guidelines [11]. Electronic databases were searched, including Embase, MEDLINE (through PubMed), Web of Science, Scopus, and Cochrane Library up to February 2022. The search terms cholangiopancreatography, endoscopic retrograde (MeSH), endoscopic retrograde cholangiopancreatography and dialysis (MeSH), or hemodialysis were used. Because there were only a few relevant studies on this topic, we included retrospective single-arm studies and comparative studies if they met the following inclusion criteria: studies on ERCP in patients on dialysis, available information on bleeding, bowel perforation, post-ERCP pancreatitis, and mortality, full-text articles, and published in English. The exclusion criteria were as follows: abstract only, reviews, case reports, commentaries, editorials, letters, irrelevancy to ERCP in renal dialysis patients, not published in English, duplicate, and incomplete data. The approval of the institutional review board (IRB) was waived in the design of this study and written consent was not needed.

### 2.2. Study Selection, Quality Assessment, and Data Extraction

Study eligibility assessments, methodological quality assessments, and data extraction were independently performed by two investigators (T.Y.P. and C.S.B.) using a standardized data form according to the predetermined selection criteria. Discrepancies between the investigators were resolved through discussions or consultations with a third evaluator (J.H.D.).

The quality of the included studies was evaluated using the risk of bias assessment tool for non-randomized studies (RoBANS) [12]. The RoBANS tool contains six domains, including the selection of participants, confounding variables, measurement of the intervention (exposure), blinding of the outcome assessment, incomplete outcome data, and selective outcome reporting [12]. RoBANS is a validated tool that is reliable and feasible for assessing the methodological quality of non-randomized studies. Review Manager version 5.3.3 (RevMan for Windows 7, the Nordic Cochrane Centre, Copenhagen, Denmark) was used to generate a summary of the RoBANS results. Data were extracted on the study design, the number of patients, demographic data, indications for ERCP, duration of dialysis, the use of anticoagulants, concomitant cirrhosis, the type of sphincter therapy, bleeding, bowel perforation, post-ERCP pancreatitis, and mortality.

### 2.3. Statistical Analysis

The primary outcome was to evaluate ERCP morbidity, including ERCP-related bleeding, post-ERCP pancreatitis, and bowel perforation, in patients on dialysis. The secondary outcome was to evaluate the mortality associated with ERCP. Comprehensive Meta-Analysis software (version 3, Biostat; Borenstein M, Hedges L, Higgins J and Rothstein H. Englewood, NJ, USA) was used for this meta-analysis. The pooled adverse event rates with 95% confidence intervals (CIs) were calculated from the enrolled studies. The heterogeneity was determined using the *I*^2^ test, developed by Higgins, and measures the percentage of the total variation across the studies [13]. *I*^2^ was calculated as follows: $I^{2}\;\left(\%\right)=100\times \left(Q-df\right)/Q$, where *Q* is Cochrane’s heterogeneity statistic and df is the degrees of freedom. Negative *I*^2^ values were set to zero, and an *I*^2^ value greater than 50% was considered to demonstrate substantial heterogeneity (range: 0–100%) [14]. Pooled effect sizes with 95% CIs were calculated using a random effects model and the DerSimonian and Laird method due to the methodological heterogeneity [15]. These results were confirmed by *I*^2^ tests. Significance was set at a *p*-value of 0.05. The publication bias was evaluated using a Begg’s funnel plot, Egger’s test of the intercept, Begg and Mazumdar’s rank correlation test, and Duval and Tweedie’s trim and fill method [16,17,18,19,20,21]. Subgroup analysis (bleeding) of specific outcomes described in all comparative studies of the dialysis group versus the non-dialysis group was also performed with pooled odds ratios (ORs) and 95% CIs using a random-effects meta-analysis. Subgroup analysis according to the type of sphincter management was performed.

## 3. Results

### 3.1. Identification of Relevant Studies

**Figure 1:**
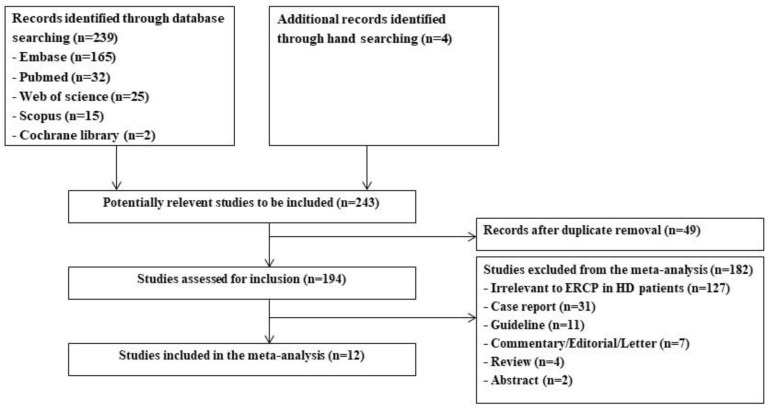
Study identification and selection process. ERCP, endoscopic retrograde cholangiopancreatography; HD, hemodialysis.

A total of 239 studies were identified from Embase (n = 165), PubMed (n = 32), Web of Science (n = 25), Scopus (n = 15), Cochrane Library (n = 2), and a manual search (n = 4). Initially, 49 duplicate studies were removed. Of the remaining 194 studies, 182 studies were excluded from the analysis for irrelevant studies on ERCP in HD patients (n = 127), case reports (n = 31), guidelines (n = 11), commentaries, editorials, and letters (n = 7), reviews (n = 4), and abstracts (n = 2). The remaining 12 studies were included in the final analysis. A flow diagram of the study identification and selection process is shown in Figure 1.

### 3.2. Characteristics of the Enrolled Publications

**Figure 2:**
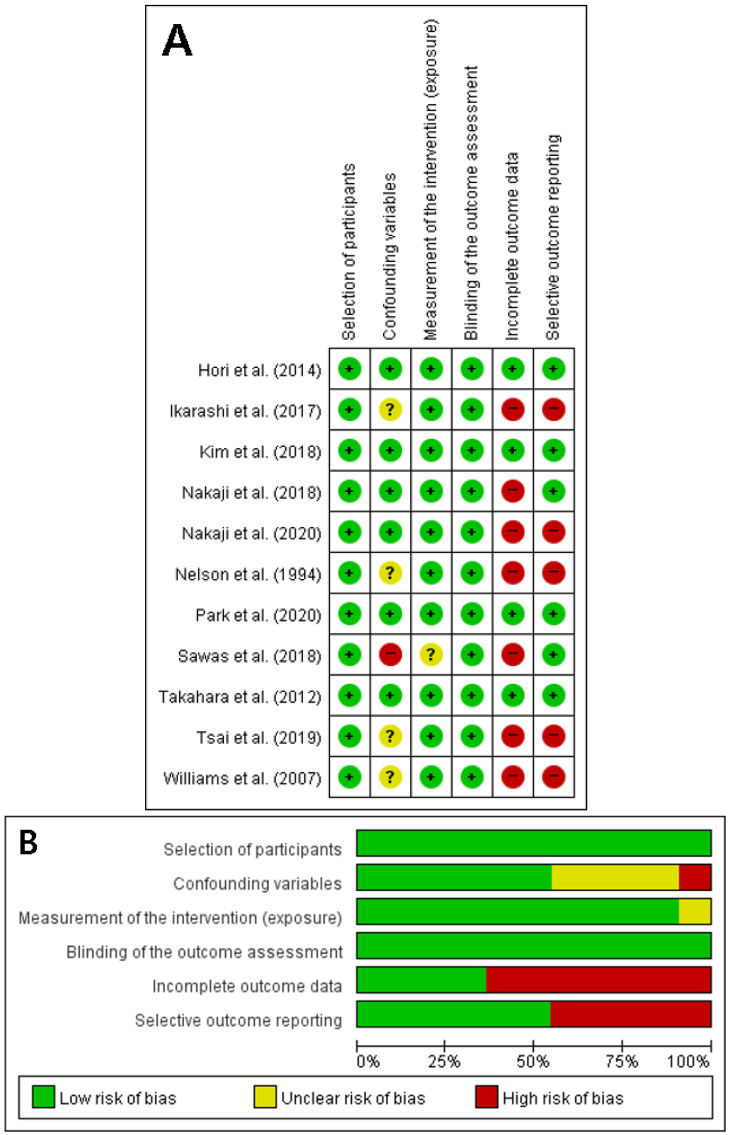
The RoBANS assessment of the methodological quality of the studies. (**A**) Detailed table. (+) denotes a low risk of bias, (?) denotes an unclear risk of bias, and (−) denotes a high risk of bias. (**B)** Summary of the RoBANS assessment. RoBANS, risk of bias assessment tool for non-randomized studies.

A total of 7921 HD patients from the 12 studies were selected for the pooled analysis, including 7 retrospective single-arm studies, 4 retrospective comparative studies, and 1 prospective single-arm study. The detailed characteristics of the included studies and patients’ demographic data are summarized in Table 1. The 12 nonrandomized studies were relatively similar in methodological quality, so a subgroup analysis was not performed. The detailed quality evaluation is described in Figure 2. Outcomes of evaluated studies are summarized in Table 2.

### 3.3. Pooled Analysis

**Figure 3:**
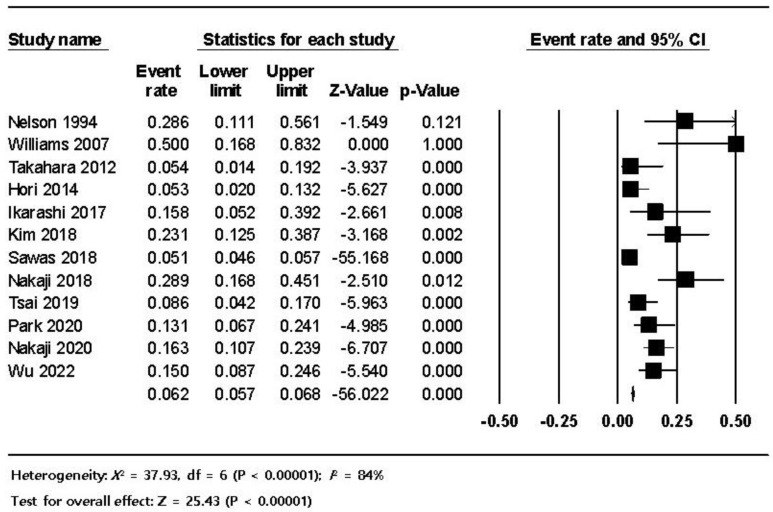
The frequency of ERCP-related bleeding in dialysis patients; the size of each square is proportional to the study’s weight. The diamond is the summary estimate. ERCP, endoscopic retrograde cholangiopancreatography; CI, confidence interval.

**Figure 4:**
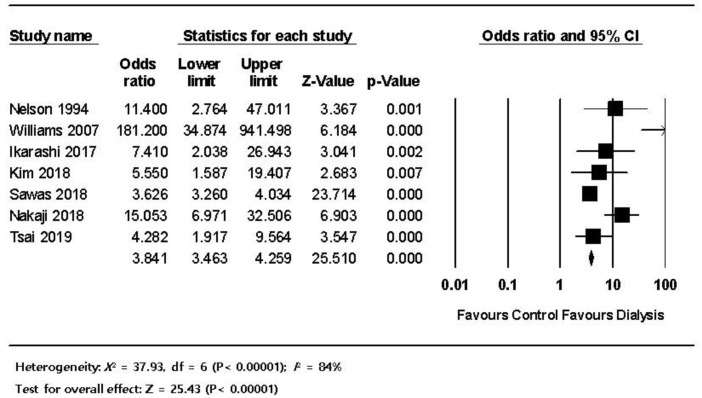
The comparison of ERCP-related bleeding in the dialysis group versus the control group; the size of each square is proportional to the study’s weight. The diamond is the summary estimate. ERCP, endoscopic retrograde cholangiopancreatography; CI, confidence interval.

**Figure 5:**
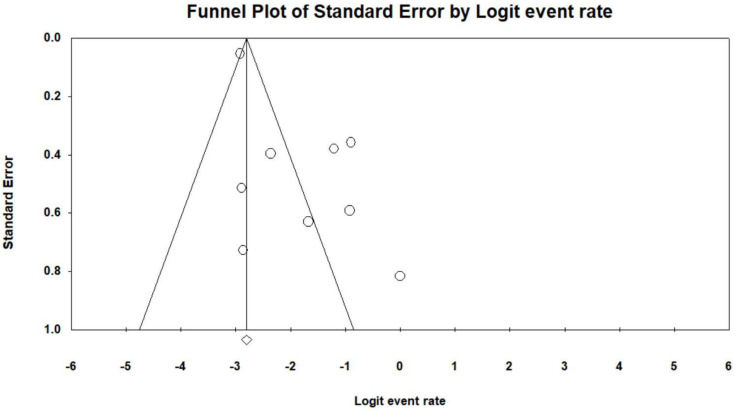
Funnel plot of bleeding in dialysis patients. The line in center is the natural logarithm of pooled event rate, and two oblique lines are pseudo 95% confidence limits. The circle means each enrolled study.

**Figure 6:**
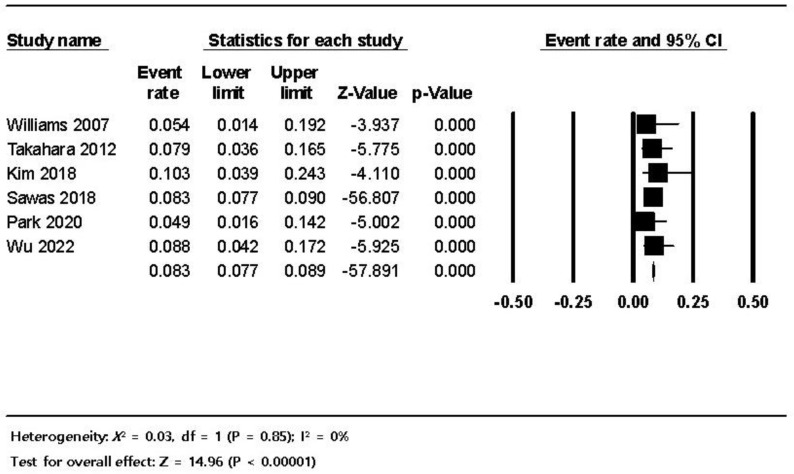
The frequency of post-ERCP pancreatitis in dialysis patients; the size of each square is proportional to the study’s weight. The diamond is the summary estimate. ERCP, endoscopic retrograde cholangiopancreatography; CI, confidence interval.

**Figure 7:**
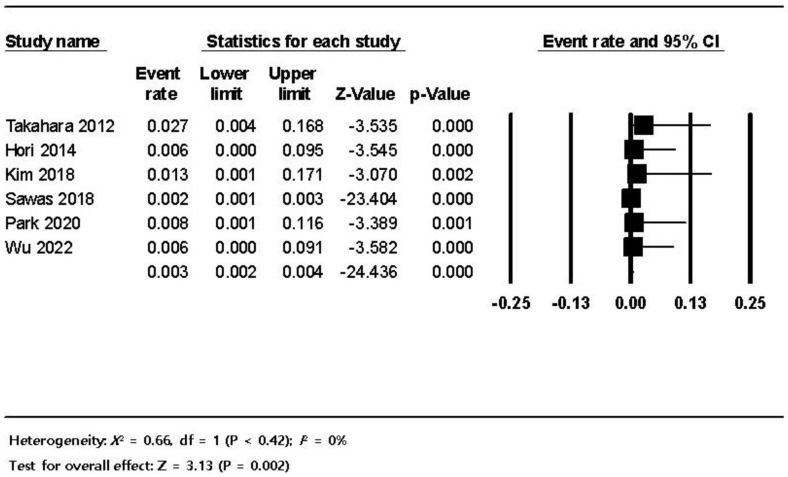
The frequency of ERCP-related bowel perforation in dialysis patients; the size of each square is proportional to the study’s weight. The diamond is the summary estimate. ERCP, endoscopic retrograde cholangiopancreatography; CI, confidence interval.

**Figure 8:**
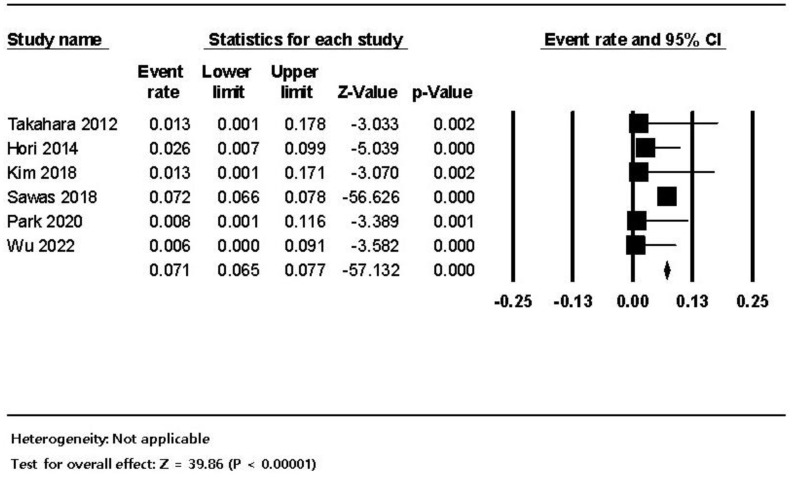
The ERCP-related mortality in dialysis patients; the size of each square is proportional to the study’s weight. The diamond is the summary estimate. ERCP, endoscopic retrograde cholangiopancreatography; CI, confidence interval.

The pooled estimated frequency of bleeding associated with ERCP from the 12 studies was 5.8% (460/7921) in the HD patients, which was higher than the 1.5% (6734/456,833) in non-HD patients. (Figure 3). In the subgroup analysis of the seven comparative studies, the ERCP-related bleeding rate was significantly higher in dialysis patients with 5.5% (414/7544) in the dialysis group versus 1.5% (6734/456,833) in the control group (OR 3.84, 95% CI 4.26–25.5, *p* < 0.001) (Figure 4). The pooled estimated frequency of bleeding was 18.4 % in the EST group, which was higher than the 12.4% in EST and/or EPBD group (Supplementary Materials: Figures S1 and S2). The funnel plot for the bleeding in dialysis patients showed asymmetry in the left lower quadrant and a trim and fill analysis showed that seven comparative studies were missed or trimmed (Figure 5). The pooled estimated frequency of post-ERCP pancreatitis was 8.3% (Figure 6). The pooled estimated frequency of bowel perforation associated with ERCP was 0. 3% (Figure 7). The pooled estimated mortality associated with ERCP was 7.1% (Figure 8). The pooled frequency of ERCP-related adverse events in the dialysis cohort is summarized in Table 3.

## 4. Discussion

This systematic review reviewed the morbidity and mortality associated with ERCP in HD patients. The pooled estimated frequency of ERCP-related bleeding was 6.2% in this review and is significantly higher than the previously reported rate of 0.3–2% in an average cohort; this increased frequency was found even though variability and severity of bleeding from post-ERCP adverse events were considered according to the definitions [5,33]. According to the subgroup analysis, a significantly higher rate of post-ERCP bleeding was observed in HD patients (5.5%, (414/7544)) compared with non-HD patients (1.5% (6734/456,833)) (OR 3.84, 95% CI 4.26–25.5, *p* < 0.001). This higher rate of post-ERCP bleeding indicates that bleeding might be a potential hazard for HD patients.

The mechanism responsible for the excessive bleeding in ESRD patients on HD has not been elucidated; however, platelet dysfunctions such as impaired platelet adhesiveness and altered platelet–vessel wall interactions are regarded as a possible reason [8]. The frequency of bleeding after endoscopic sphincterotomy (EST) in HD patients has been reported to be 8.1–23.1% [27,29] and is higher than in the general population, which is 2.5–5% [5]. In a recent study, the frequency of post-EST bleeding was reported to be up to 29% in ESRD patients on HD, and this risk is significantly higher than in non-HD patients (OR, 13.30; 95 % CI, 5.78–30.80; *p <* 0.001) [28]. Endoscopic papillary balloon dilation (EPBD) can be used as an alternative to EST in cases with a high risk of bleeding, such as liver cirrhosis or coagulopathy patients, to avoid bleeding. However, some reports have indicated the high risk of post-ERCP bleeding when performing ERCP in patients receiving anticoagulant agents [34,35]. Recently, post-ERCP bleeding was compared the different sphincter management techniques, EST, EPBD, and one-half EST plus EPBD, in ESRD patients on HD. One-half EST followed by EPBD combination therapy significantly reduced post-ERCP bleeding compared with EST single therapy (OR, 0.07; 95% CI, 0.01–0.72; *p* = 0.026) [32].

Although evaluations of ERCP-related adverse events in HD patients are an important clinical issue, prospective randomized controlled trials regarding the clinical outcomes of ERCP in HD patients and the general population have never been conducted because of practical limitations. However, several retrospective studies from 2012 have been reported, but during the ten years until now, a total of 10 retrospective full-text papers have been published. The first retrospective observatory study regarding ERCP using an HD cohort was published in 2012 and evaluated EPBD on HD patients instead of EST as a sphincter management method [24]. The authors concluded that EPBD needed to be performed carefully in HD patients with an additional bleeding risk factor, such as Child-Pugh class C liver cirrhosis or those taking antiplatelet agents at the time of the EPBD [36].

Recently, a predictive model of bleeding following EST was created using cut-off values for age, platelet count, prothrombin time and INR (PT-INR), and the duration of HD, which increased the post-EST bleeding risk, and used data from 123 HD patients [31]. In HD patients, a platelet count of less than 120,00 was a strong risk factor for post-EST bleeding.

Post-ERCP pancreatitis is the most common adverse event and is always a worrisome issue because it can result in considerable morbidity and mortality in HD patients as well as the general population [37,38,39]. In the general population, the frequency of post-ERCP pancreatitis has been reported to be 3.4% to 6.0% in average-risk groups and 8% to 13.1% in high-risk groups [40]. In HD patients, the frequency of post-ERCP pancreatitis has been reported to be 5.4% to 10.3%, which is similar to the general population [10,24,25,27]. However, acute pancreatitis in HD patients is more of a worry because of the vigorous fluid therapy, which is the primary treatment for acute pancreatitis, and HD would be restricted due to volume overloading.

The pathogenesis of post-ERCP pancreatitis has not been established, and it seems to be related to whether the uremic condition of HD patients affects the development or prevention of post-ERCP pancreatitis. In addition, the prevention of post-ERCP pancreatitis has not been determined even though, in the general population, the administration of rectal non-steroidal anti-inflammatory drugs (NSAIDs) has been known to prevent post-ERCP pancreatitis. However, the effect of rectal NSAIDs varies and is suboptimal according to the patient’s post-ERCP pancreatitis risk, ERCP technique, the use of a prophylactic pancreatic duct stent, and other pharmacologic modalities. In HD patients, the use of rectal NSAIDs is limited and is commercially unavailable in some countries. Prophylactic fluid therapy is also limited due to volume overloading, pulmonary edema, or cardiac congestion in HD patients. Although, prophylactic pancreatic duct stent insertion can be performed regardless of the patient’s condition.

The risk of bowel perforation is high in HD patients due to vulnerable bowels. There are exceptionally high risks of bowel perforation in peritoneal dialysis (PD) patients due to underlying intra-abdominal adhesions and peritonitis. In a large retrospective study, the mortality of ERCP was reported to be 7.1% in patients with ESRD, which was significantly higher than in patients without ESRD (1.2%) [10]. The frequency of perforation during ERCP was reported to be approximately 0.1–1.0% [5,6,41]. In this meta-analysis, the pooled estimated frequency of perforation associated with ERCP was 0.6%, which is comparable with the previous studies [5,6,41]. Perforation, such as EST site perforation or free duodenal perforation, is a rare complication of ERCP, but depending on the anatomic site of the perforation, surgical interventions, such as Whipple’s operation or pylorus-preserving pancreaticoduodenectomy (PPPD), can be required; however, these interventions are associated with a mortality of 8.5–32% [42,43]. However, in cases where the perforation occurred due to duodenoscopy, the perforation can be fixed with minor surgery, such as over the scope clips (OTSCs), unlike PPPD [44,45,46]. Therefore, PPPD after ERCP perforation is extremely rare.

The mortality associated with ERCP has been reported to be 0.8–2.2% in previous studies [5]. The pooled estimate of ERCP-related mortality in this study was 4.1%, which is higher than that in the previous reports. Two studies observed mortality out of the four included in this meta-analysis [10,24,25,27]. One of the two is a small observational noncomparative study from a Japanese HD cohort [25]. The mortality rate in this study was 2.6% (2/76), and the deaths were caused by post-ERCP pancreatitis and aspiration pneumonia. The other is a large retrospective case-control study from a nationwide inpatient sample (NIS) from a United States cohort [10]. The mortality of ERCP in patients with ERSD in this study was significantly higher than that in patients without renal dysfunction (7.1% (526/7347) vs. 1.2% (5138/445,424), OR 3.7, 95% CI 2.9–4.6, *p* < 0.001) [10].

This meta-analysis has several notable inherent limitations. First, due to a scarcity of relevant, high-quality studies, this meta-analysis is mainly based on a few low-quality studies, including noncomparative retrospective studies. Clinical trials evaluating the outcomes of ERCP in patients with ESRD would be unethical and clinically problematic since performing ERCP on high-risk patients could result in procedure-related morbidity and mortality. Because of this, studies regarding this issue are scarce, and inevitably retrospective single-arm studies were included in the final analysis; therefore, conclusions are limited. Second, 7921 HD patients from 12 studies were included; however, 7347 (92.8%) of the patients were from one study [10]. Therefore, the result of this analysis is strongly dependent on only one study and is a severe limitation of this pooled analysis. Third, there are many conflicting factors in performing ERCP in ESRD patients, and none of these factors could be considered in this meta-analysis, including heterogeneous indications for ERCP, the use of anticoagulants, underlying cardiopulmonary diseases, duration of hemodialysis, the type of sphincter management, such as EST, EPBD, or endoscopic papillary large balloon dilation (EPLBD), rescue precut or fistulotomy, prophylactic pancreatic stents or pharmacologic agents for prevention of post-ERCP pancreatitis, the experience of the endoscopist, the hospital volume of ERCP procedures, periampullary diverticulum, and the use of magnetic resonance cholangiopancreatography (MRCP) as an alternative diagnostic to ERCP. Fourth, subgroup analysis of important factors for post ERCP-bleeding including the use of anticoagulants, concomitant cirrhosis, the type of sphincter therapy did not performed due to incomplete data. This can be notable limitation of this systematic review.

In conclusion, this pooled analysis showed that ERCP-related morbidity and mortality are higher in HD patients than in non-dialysis patients. Mainly, the subgroup analysis identified that a significantly higher rate of post-ERCP bleeding was observed in HD patients. Therefore, considering the high risk of procedure-related adverse events, further attention should be given prior to performing ERCP on HD patients, and additional risk versus benefit assessments should be performed before making decisions regarding ERCP in HD patients.

## Figures and Tables

**Table 1:** Characteristics of the studies included in the meta-analysis.

Study	Nation	Design	No. of Patients	Indications for ERCP, No.	Duration of Dialysis, Years	Anticoagulant Use, No.	Cirrhosis, No.
Nelson [22], 1994	United States	Retrospective single arm	HD 14 Non-dialysis 177	CBD stone 73 Cholangitis 41 Tumor/stricture 26 Gallstone pancreatitis 16 SOD/papillary stenosis 17 Bile leak 4 Others 4	*N/A*	5	*N/A*
Williams [23], 2007	United Kingdom	Prospective single arm	HD 6 Non-dialysis 4555	CBD stone 2477 Malignancy 891 Pancreatitis 435 Gallstone pancreatitis 16 Cholangitis 245 Bile leak 100 SOD 69 Others 344	*N/A*	*N/A*	43
Takahara [24], 2012	Japan	Retrospective single arm	HD 37	CBD stone 37	5.1 (0.03–14.8) *	12	6
Hori [25], 2014	Japan	Retrospective single arm	HD 76	CBD stone 43 Mucinous neoplasm 7 Pancreas cancer 6 Others 20	6 (1–35) *	30	2
Ikarashi [26], 2017	Japan	Retrospective single arm	HD 19 Non-dialysis 1094	CBD stone 801 Malignant biliary stricture 256 Sphincter of Oddi dysfunction 13 Acute cholecystitis 11 Benign biliary stricture 11 Other 21	*N/A*	56	16
Kim [27], 2018	Republic of Korea	Retrospective comparative	HD 28, PD 11 Non-dialysis 78	CBD stone 117	8 (1–24) *	13 12	1 2
Sawas [10], 2018	United States	Retrospective comparative	ESRD 7347 CKD 39,403 Non-dialysis 445,424	*N/A*	*N/A*	*N/A*	*N/A*
Nakaji [28], 2018	Japan	Retrospective comparative	HD 38 Non-dialysis 1480	CBD stone 1518	*N/A*	25 1	12
Tsai [29], 2019	Taiwan	Retrospective comparative	HD 74 Non-dialysis 3487	CBD stone 64 Malignancy 9 Pancreatitis 5	*N/A*	None **	None **
Park [30], 2020	Republic of Korea	Retrospective single arm	HD 61	CBD stone 61	4.1 (4.7) †	38	*N/A*
Nakaji [31], 2020	Japan	Retrospective single arm	HD 123	CBD stone 123	5 (1–24) *	8	*0*
Wu [32], 2022	Taiwan	Retrospective single arm	HD 80	CBD stone 80	*N/A*	31	*N/A*

ERCP, endoscopic retrograde cholangiopancreatography; HD, hemodialysis; PD, peritoneal dialysis; ESRD, end-stage renal disease; CKD, chronic kidney disease; CBD, common bile duct; SOD, sphincter of Oddi dysfunction; *N/A*, not available. * Median, (Range); † Mean, (SD); ** Patients with anticoagulant use and cirrhosis were excluded from this study.

**Table 2:** Outcomes of evaluated studies.

Study	Groups	No. of Patients (Procedures)	Age, Years	Sex, M:F	Type of Sphincter Therapy	Bleeding, No. (%)	Post-ERCP Pancreatitis, No. (%)	Bowel Perforation, No. (%)	Mortality, No. (%)
Nelson [22], 1994	Dialysis Control	14 177	66 (19)	108:69	EST	4/14 (28.6) 6/177 (3.4)	*N/A*	*N/A*	*N/A*
Williams [23], 2007	Dialysis Control	6 4555	65.0 (16.7)	1970:2591	EST	3/6 (50) 25/4555	*N/A*	*N/A*	*N/A*
Takahara [24], 2012	Dialysis	37	71 (49–88) *	25:12	EPBD	2/37 (5.4)	2/37 (5.4)	1/37 (2.7)	0/37 (0)
Hori [25], 2014	Dialysis	76	70 (33–87) *	55:21	EST ± EPBD	4/76 (5.3)	6/76 (7.9)	0/76 (0)	2/76 (2.9)
Ikarashi [26], 2017	Dialysis Control	19 1094	74 (14–101) *	643:470	EST	3/19 (15.8) 27/1094 (2.5)	*N/A*	*N/A*	*N/A*
Kim [27], 2018	Dialysis Control	39 78	65.6 (12.6) † 65.6 (12.5) †	22:17 46/32	EST or EPBD ± NF	9/39 (23.1) 4/78 (5.1)	4/39 (10.3) 5/78 (6.4)	0/39 (0) 1/78 (1.3)	0/39 (0) 0/78 (0)
Sawas [10], 2018	Dialysis Control	7347 445,424	65.5 (0.42) † 58 (0.12) †	3870:3477 174,124:271,300	*N/A*	377/7347 (5.1) 6546/445,424 (1.5)	611/7347 (8.3) 20,315/445,424 (4.6)	14/7347 (0.2) 340/445,424 (0.07)	526/7347 (7.1) 5138/445,424 (1.15)
Nakaji [28], 2018	Dialysis Control	38 1480	70.7 (9.3) † 74.8 (12.9) †	22:16 791:689	EST ± EPLBD	11/38 (29.0) 39/1480 (2.6)	*N/A*	*N/A*	*N/A*
Tsai [29], 2019	Dialysis Control	74 (81) 3487 (4025)	67 (33–86) **	32:42	EST or EPBD	** 7/81 (8.64) ** 87/4025 (2.16)	*N/A*	*N/A*	*N/A*
Park [30], 2020	Dialysis	61	69.7 (10.7) †	36: 25	EST 30 EPBD 23 EST + EPBD 8	8/61(13.1)	3/61 (4.9)	0/61 (0)	0/61 (0)
Nakaji [31], 2020	Dialysis	123	71(47–101) *	79:44	EST ± EPLBD	20/123 (16.3)	*N/A*	*N/A*	*N/A*
Wu [32], 2022	Dialysis	80	*N/A*	36:44	EST 21 EPBD 28 EST + EPBD 31	12/80 (15.0)	7/80 (8.8)	0/80 (0)	0/80 (0)

ERCP, endoscopic retrograde cholangiopancreatography; *N/A*, not available; EST, endoscopic sphincterotomy; NK, needle knife; EPBD, endoscopic papillary balloon dilation; EPLBD, endoscopic papillary large balloon dilation. CKD patients without dialysis were excluded from the final meta-analysis. * Median, (Range). † Mean, (SD). ** Events/procedures.

**Table 3:** Summary of ERCP-related adverse events in 7921 dialysis patients.

Adverse Events	No. (%)
Post-ERCP pancreatitis *	633 (8.3)
Mortality *	528 (7.1)
Bleeding	460 (5.8)
Bowel perforation *	15 (0.3)

ERCP, endoscopic retrograde cholangiopancreatography. * Total number (n = 7640).

## Data Availability

Not applicable.

## Supplementary Materials

The following supporting information can be downloaded at: https://www.mdpi.com/article/10.3390/jpm12111883/s1, Figure S1: The frequency of ERCP-related bleeding in HD patients with EST. Figure S2: The frequency of ERCP-related bleeding in HD patients with EST and/or EPBD.

## References

[1] Hahm J.S., Lee H.L., Park J.Y., Eun C.S., Han D.S., Choi H.S. (2003). Prevalence of gallstone disease in patients with end-stage renal disease treated with hemodialysis in Korea. Hepatogastroenterology.

[2] Badalamenti S., DeFazio C., Castelnovo C., SanGiovanni A., Como G., De Vecchi A., Graziani G., Colombo M., Ponticelli C. (1994). High Prevalence of Silent Gallstone Disease in Dialysis Patients. Nephron Exp. Nephrol..

[3] Kazama J.J., Kazama S., Koda R., Yamamoto S., Narita I., Gejyo F. (2009). The Risk of Gallbladder Stone Formation Is Increased in Patients with Predialysis Chronic Kidney Disease but Not Those Undergoing Chronic Hemodialysis Therapy. Nephron Clin. Pract..

[4] Naitoh I., Hori Y. (2018). Post-ERCP Complications in Dialysis Patients: Cutting One’s Losses or Expanding Possibilities?. Dig. Dis. Sci..

[5] Cotton P., Lehman G., Vennes J., Geenen J., Russell R., Meyers W., Liguory C., Nickl N. (1991). Endoscopic sphincterotomy complications and their management: An attempt at consensus. Gastrointest. Endosc..

[6] Cotton P.B., Garrow D.A., Gallagher J., Romagnuolo J. (2009). Risk factors for complications after ERCP: A multivariate analysis of 11,497 procedures over 12 years. Gastrointest. Endosc..

[7] Sabovic M., Salobir B., Zupan I.P., Bratina P., Bojec V., Ponikvar J.B. (2005). The Influence of the Haemodialysis Procedure on Platelets, Coagulation and Fibrinolysis. Pathophysiol. Haemost. Thromb..

[8] Eberst M.E., Berkowitz L.R. (1994). Hemostasis in renal disease: Pathophysiology and management. Am. J. Med..

[9] Zuckerman G.R., Cornette G.L., Clouse R.E., Harter H.R. (1985). Upper Gastrointestinal Bleeding in Patients with Chronic Renal Failure. Ann. Intern. Med..

[10] Sawas T., Bazerbachi F., Haffar S., Cho W.K., Levy M.J., A Martin J., Petersen B.T., Topazian M.D., Chandrasekhara V., Abu Dayyeh B.K. (2018). End-stage renal disease is associated with increased post endoscopic retrograde cholangiopancreatography adverse events in hospitalized patients. World J. Gastroenterol..

[11] Moher D., Liberati A., Tetzlaff J., Altman D.G., PRISMA Group (2009). Preferred reporting items for systematic reviews and meta-analyses: The PRISMA statement. BMJ.

[12] Kim S.Y., Park J.E., Lee Y.J., Seo H.J., Sheen S.S., Hahn S., Jang B.H., Son H.J. (2013). Testing a tool for assessing the risk of bias for nonrandomized studies showed moderate reliability and promising validity. J. Clin. Epidemiol..

[13] Higgins J.P.T., Thompson S.G. (2002). Quantifying heterogeneity in a meta-analysis. Stat. Med..

[14] Higgins J.P.T., Thompson S.G., Deeks J.J., Altman D.G. (2003). Measuring inconsistency in meta-analyses. BMJ.

[15] DerSimonian R., Laird N. (1986). Meta-analysis in clinical trials. Control. Clin. Trials.

[16] Duval S., Tweedie R. (2000). Trim and Fill: A Simple Funnel-Plot-Based Method of Testing and Adjusting for Publication Bias in Meta-Analysis. Biometrics.

[17] Sutton A.J.A.K., Jones D.R., Sheldon T.A., Song F. (2000). Methods for Meta-Analysis in Medical Research.

[18] Sterne J.A., Egger M. (2001). Funnel plots for detecting bias in meta-analysis: Guidelines on choice of axis. J. Clin. Epidemiol..

[19] Begg C.B., Mazumdar M. (1994). Operating Characteristics of a Rank Correlation Test for Publication Bias. Biometrics.

[20] Egger M., Smith G.D., Schneider M., Minder C. (1997). Bias in meta-analysis detected by a simple, graphical test. BMJ.

[21] Higgins J.P.G.S. (2011). Cochrane Handbook for Systemic Reviews of Interventions.

[22] Nelson D.B., Freeman M.L. (1994). Major Hemorrhage from Endoscopic Sphincterotomy: Risk Factor Analysis. J. Clin. Gastroenterol..

[23] Williams E., Taylor S., Fairclough P., Hamlyn A., Logan R., Martin D., Riley S., Veitch P., Wilkinson M., Williamson P. (2007). Risk factors for complication following ERCP; results of a large-scale, prospective multicenter study. Endoscopy.

[24] Takahara N., Isayama H., Sasaki T., Tsujino T., Toda N., Sasahira N., Mizuno S., Kawakubo K., Kogure H., Yamamoto N. (2012). Endoscopic papillary balloon dilation for bile duct stones in patients on hemodialysis. J. Gastroenterol..

[25] Hori Y., Naitoh I., Nakazawa T., Hayashi K., Miyabe K., Shimizu S., Kondo H., Yoshida M., Yamashita H., Umemura S. (2014). Feasibility of endoscopic retrograde cholangiopancreatography-related procedures in hemodialysis patients. J. Gastroenterol. Hepatol..

[26] Ikarashi S., Katanuma A., Kin T., Takahashi K., Yane K., Sano I., Yamazaki H., Maguchi H. (2017). Factors associated with delayed hemorrhage after endoscopic sphincterotomy: Japanese large single-center experience. J. Gastroenterol..

[27] Kim S.B., Kim K.H., Kim T.N. (2018). Safety and Efficacy of Endoscopic Retrograde Cholangiopancreatography for Choledocholithiasis in Long-Term Dialysis: A Propensity Score Analysis. Am. J. Dig. Dis..

[28] Nakaji S., Hirata N., Matsui H., Shiratori T., Kobayashi M., Yoshimura S., Kanda K., Kawamitsu N., Harasawa H. (2018). Hemodialysis is a strong risk factor for post-endoscopic sphincterotomy bleeding in patients with choledocholithiasis. Endosc. Int. Open.

[29] Tsai M.C., Wang C.C., Wang Y.T., Yang T.W., Chen H.Y. (2019). Major bleeding risk of endoscopic sphincterotomy versus endoscopic papillary balloon dilatation in hemodialysis patients. Saudi J. Gastroenterol..

[30] Park J.S., Jeong S., Cho J.H., Kwon C.I., Jang S.I., Lee T.H., Han J.H., Hwang J.C., Lee D.H. (2020). Clinical outcome of endoscopic retrograde cholangiopancreatography for choledocholithiasis in end-stage renal disease patients on hemodialysis. Turk. J. Gastroenterol..

[31] Nakaji S., Okawa Y., Nakamura K., Itonaga M., Inase M., Sugiyama H., Suzuki R., Yamauchi K., Matsui H., Hirata N. (2020). Predictive model of bleeding following endoscopic sphincterotomy for the treatment of choledocholithiasis in hemodialysis patients: A retrospective multicenter study. JGH Open.

[32] Wu J.H., Kang J.W., Wang Y.S., Lin H.J., Chen C.Y. (2022). Comparison of Different Endoscopic Methods Used for Managing Choledocholithiasis in Patients with End-Stage Renal Disease Undergoing Hemodialysis. Am. J. Dig. Dis..

[33] Chandrasekhara V., Khashab M.A., Muthusamy V.R., Acosta R.D., Agrawal D., Bruining D.H., Eloubeidi M.A., Fanelli R.D., Faulx A.L., Gurudu S.R. (2016). Adverse events associated with ERCP. Gastrointest. Endosc..

[34] Hamada T., Yasunaga H., Nakai Y., Isayama H., Matsui H., Horiguchi H., Fushimi K., Koike K. (2015). Bleeding after endoscopic sphincterotomy or papillary balloon dilation among users of antithrombotic agents. Endoscopy.

[35] Fujimoto K., Fujishiro M., Kato M., Higuchi K., Iwakiri R., Sakamoto C., Uchiyama S., Kashiwagi A., Ogawa H., Murakami K. (2013). Guidelines for gastroenterological endoscopy in patients undergoing antithrombotic treatment. Dig. Endosc..

[36] Kawabe T., Komatsu Y., Tada M., Toda N., Ohashi M., Shiratori Y., Omata M. (1996). Endoscopic Papillary Balloon Dilation in Cirrhotic Patients: Removal of Common Bile Duct Stones without Sphincterotomy. Endoscopy.

[37] Freeman M.L., Nelson D.B., Sherman S., Haber G.B., Herman M.E., Dorsher P.J., Moore J.P., Fennerty M.B., Ryan M.E., Shaw M.J. (1996). Complications of Endoscopic Biliary Sphincterotomy. N. Engl. J. Med..

[38] Freeman M.L., Guda N.M. (2004). Prevention of post-ERCP pancreatitis: A comprehensive review. Gastrointest. Endosc..

[39] Kochar B., Akshintala V.S., Afghani E., Elmunzer B.J., Kim K.J., Lennon A.M., Khashab M.A., Kalloo A.N., Singh V.K. (2015). Incidence, severity, and mortality of post-ERCP pancreatitis: A systematic review by using randomized, controlled trials. Gastrointest. Endosc..

[40] Park T.Y., Oh H.C., Fogel E.L., Lehman G.A. (2020). Prevention of post-endoscopic retrograde cholangiopancreatography pancreatitis with rectal non-steroidal anti-inflammatory drugs. Korean J. Intern. Med..

[41] Masci E., Toti G., Mariani A., Curioni S., Lomazzi A., Dinelli M., Minoli G., Crosta C., Comin U., Fertitta A. (2001). Complications of diagnostic and therapeutic ERCP: A prospective multicenter study. Am. J. Gastroenterol..

[42] Jin Y.J., Jeong S., Kim J.H., Hwang J.C., Yoo B.M., Moon J.H., Park S.H., Kim H.G., Lee D.K., Jeon Y.S. (2013). Clinical course and proposed treatment strategy for ERCP-related duodenal perforation: A multicenter analysis. Laryngo-Rhino-Otologie.

[43] Patil N.S., Solanki N., Mishra P.K., Sharma B.C., Saluja S.S. (2019). ERCP-related perforation: An analysis of operative outcomes in a large series over 12 years. Surg. Endosc..

[44] Kirtane T., Singhal S. (2015). Endoscopic closure of iatrogenic duodenal perforation using dual over-the-scope clips. Gastrointest. Endosc..

[45] Di Saverio S., Segalini E., Birindelli A., Todero S., Podda M., Rizzuto A., Tugnoli G., Biondi A. (2018). Pancreas-sparing, ampulla-preserving duodenectomy for major duodenal (D1-D2) perforations. Br. J. Surg..

[46] Iwasa Y., Iwashita T., Uemura S., Mita N., Iwata K., Yoshida K., Mukai T., Yasuda I., Shimizu M. (2020). The Efficacy of Over-the-Scope Clip Closure for Gastrointestinal Iatrogenic Perforation During Endoscopic Ultrasound and Endoscopic Retrograde Cholangiopancreatography for Pancreaticobiliary Diseases. Surg. Laparosc. Endosc. Percutaneous Tech..

