# Supplementary material for: Outcomes of Endoscopic Retrograde Cholangiopancreatography in End-Stage Renal Disease Patients Undergoing Hemodialysis: A Systematic Review and Pooled Analysis
Source: J Pers Med. 2022 Nov 10;12(11):1883. doi: 10.3390/jpm12111883 (PMC9697903; doi:10.3390/jpm12111883)

Supplementary Materials, Figure S1: The frequency of ERCP-related bleeding in HD patients with EST. The size of each square is proportional to the study's weight. The diamond is the summary estimate. ERCP, endoscopic retrograde cholangiopancreatography; EST, endoscopic sphincterotomy; HD, hemodialysis; CI, confidence interval.

Study name	Statistics for each study				
	Event rate	Lower limit	Upper limit	Z-Value	p-Value
Nelson et al. (1994)	0.286	0.111	0.561	-1.549	0.121
Williams et al. (2007)	0.500	0.168	0.832	0.000	1.000
Ikarashi et al. (2017)	0.158	0.052	0.392	-2.661	0.008
Sawas et al. (2018)	0.051	0.046	0.057	-55.168	0.000
	0.184	0.051	0.485	-2.040	0.041

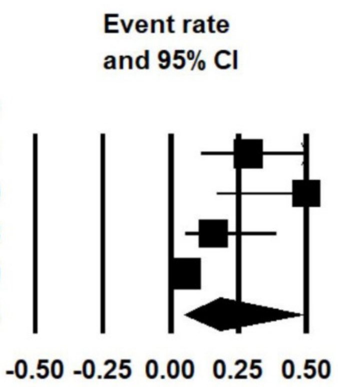

Supplementary Materials, Figure S2: The frequency of ERCP-related bleeding in HD patients with EST and/or EPBD. The size of each square is proportional to the study's weight. The diamond is the summary estimate. ERCP, endoscopic retrograde cholangiopancreatography; HD, hemodialysis; EST, endoscopic sphincterotomy; EPBD, endoscopic papillary balloon dilation; CI, confidence interval.

Study name	Statistics for each study				
	Event rate	Lower limit	Upper limit	Z-Value	p-Value
Takahara et al. (2012)	0.054	0.014	0.192	-3.937	0.000
Hori et al. (2014)	0.053	0.020	0.132	-5.627	0.000
Kim et al. (2018)	0.231	0.125	0.387	-3.168	0.002
Nakaji et al. (2018)	0.289	0.168	0.451	-2.510	0.012
Tsai et al. (2019)	0.086	0.042	0.170	-5.963	0.000
	0.124	0.059	0.243	-4.673	0.000

Event rate and 95% CI

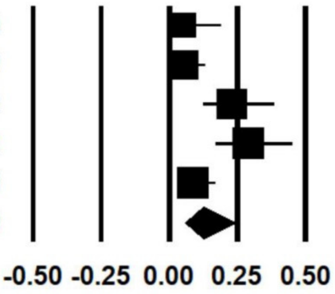

Supplement: Supplementary file 1 [file jpm-12-01883-s001.zip › jpm-1886737-supplementary.pdf]
